# Successful resolution of ectopic Cushing syndrome by minimally invasive thoracoscopic resection of the neuroendocrine tumor of the thymus: a rare case report

**DOI:** 10.1186/s12893-022-01674-0

**Published:** 2022-06-11

**Authors:** Zizi Zhou, Wenxiang Chai, Longhai Yang, Yi Liu, Yao Liu, Huiyu Pan, Qiang Wu, Xiaoming Zhang, Eric Dominic Roessner

**Affiliations:** 1 Department of Cardio-Thoracic Surgery, Shenzhen University General Hospital, Xueyuan Avenue 1098, Nanshan District, 518055 Shenzhen, China; 2 Department of Thoracic Surgery, University Medical Center Mainz, Johannes Gutenberg University Mainz, Mainz, Germany

**Keywords:** Cushing syndrome, Hyperaldosteronism, Thoracoscopic surgery, Neuroendocrine tumors, Case report

## Abstract

**Background:**

Ectopic Cushing syndrome (ECS) is a sporadic condition. Even uncommon is an ECS that derives from a carcinoid tumor of the thymus. These tumors may pose several diagnostic and therapeutic conundrums. This report discusses the differential diagnosis, clinicopathological findings, and effective treatment of a rare case of ECS using a minimally invasive approach.

**Case presentation:**

A 29-year-old woman with Cushing syndrome presented with facial flushing. Physical examination revealed hypertension (blood pressure: 141/100 mmHg). A mediastinal tumor was discovered to be the cause of the patient’s chronic hypokalemia and hypercortisolemia. Cortisol levels increased in the morning, reaching 47.7 ug/dL. The levels of the hormones ACTH, aldosterone, and renin were determined to be 281 pg/mL, 3.0 ng/dL, and 2.1 pg/mL, respectively. The presence of hypertension, hypokalemia, and alkalinity suggested Cushing’s syndrome, which was proven to be ACTH-dependent ECS by a dexamethasone suppression test. A chest CT scan revealed inflammation in the posterior basal region of the right lower lobe. The superior anterior mediastinum was characterized by round-shaped isodensity lesions with distinct borders. She underwent thoracoscopic anterior mediastinal tumor excision via the subxiphoid technique (R0 resection); following surgery, her blood pressure returned to normal, and the hypernatremia/hypopotassemia resolved. The tumor was determined to be a thymic carcinoid. Most notably, cortisol levels fell to half of their presurgical levels after one hour of surgery, and other abnormalities corrected substantially postoperatively.

**Conclusion:**

Thoracoscopic excision of thymic tumors by subxiphoid incision may be a useful treatment option for ECS caused by neuroendocrine tumors of the thymus

## Core tip

Cushing syndrome is caused by chronic exposure to an excess of endogenous or exogenous glucocorticoids that affect the normal function of the hypothalamic-pituitary-adrenal axis. Ectopic Cushing Syndrome (ECS) is a rare and complicated condition with serious morbidity concerns. A female patient was diagnosed with ECS caused by a thymus neuroendocrine tumor, an extremely rare cause of ECS. The single subxiphoid incision could be a novel approach in this setting; however, to the best of our knowledge, it has not been employed in the context of ECS involving anterior mediastinum tumors. After a comprehensive diagnostic assessment, we could successfully manage this complicated case by thoracoscopic resection via a single subxiphoid incision, demonstrating that if there are no metastases, thoracoscopic resection via the subxiphoid incision of the whole lesion can be effectively performed.

## Background

Cushing syndrome (CS) affects 2–25 people per million in the general population each year [1]. It is primarily a consequence of prolonged exposure to endogenous or exogenous excess of glucocorticoids, which disrupts the normal hypothalamic-pituitary-adrenal axis [2]. The most frequent foundation for the categorization of CS is the dependence on adrenocorticotropic hormone (ACTH) [3]. ACTH-dependent CS is most commonly caused by a pituitary corticotrophic adenoma; however, it can also be driven by an extra pituitary tumor [4]. Ectopic CS (ECS) is very uncommon, with just one instance per million people per year [2]. ECS, unlike pituitary-dependent CS, has a rapid onset and is associated with weight loss, muscular weakness, hypokalemic alkalosis, and elevated serum cortisol [5]. It is primarily responsible for endogenous hypercortisolism, which accounts for 5–20% of all incidences of CS [6]. In most cases with ECS, there is an apparent underlying tumor [7, 8]. Moreover, even comparatively benign tumors, such as carcinoid tumors with slow growth, may cause a clinical condition that is similar to pituitary CS [6, 9, 10]. These tumors generate a variety of physiologically active substances; nevertheless, determining the ectopic source of ACTH and treating hypercortisolism are challenging diagnostic and therapeutic undertakings [11].

Neuroendocrine tumors pose unique diagnostic and treatment difficulties in this setting due to their ability to form at a variety of anatomical sites. Patients with these types of tumors frequently suffer an elevated risk of morbidity as a result of diagnostic and therapeutic delays. Because of advancements in minimally invasive technology, the thoracoscopic method has grown more common among other treatment modalities in recent years. The single subxiphoid incision is a novel way to perform such operations; however, to the best of our knowledge, it has not been employed in the context of ECS involving anterior mediastinum tumors [12, 13]. The current study describes a rare case in which an ECS patient was effectively treated with thoracoscopic surgery, resulting in rapid improvement in the clinical symptoms. The patient was diagnosed to have a small atypical carcinoid tumor. The tumor was successfully reselected using a subxiphoid technique and minimally invasive thoracoscopic surgery, leading to CS resolution.

## Case presentation

### Chief complaints

The primary symptoms were face flushing for one month and hypertension for one day.

### History of present illness

A 29-year-old non-smoking and non-alcoholic woman was admitted. A functional examination showed no mood swings, impatience, dread, tiredness, hunger, polyphagia, palpitation, or paroxysmal sweating. There was no apparent impairment in vision, chest discomfort, fever, cough, or cough, and sputum throughout the course of the disease.

### History of past illness

She had a history of polycystic ovary syndrome diagnosed five years ago.

### Personal and family history

The patient had a normal obstetric history and no significant family or surgical history. Her social history was also normal.

### Physical examination

**Fig. 1:**
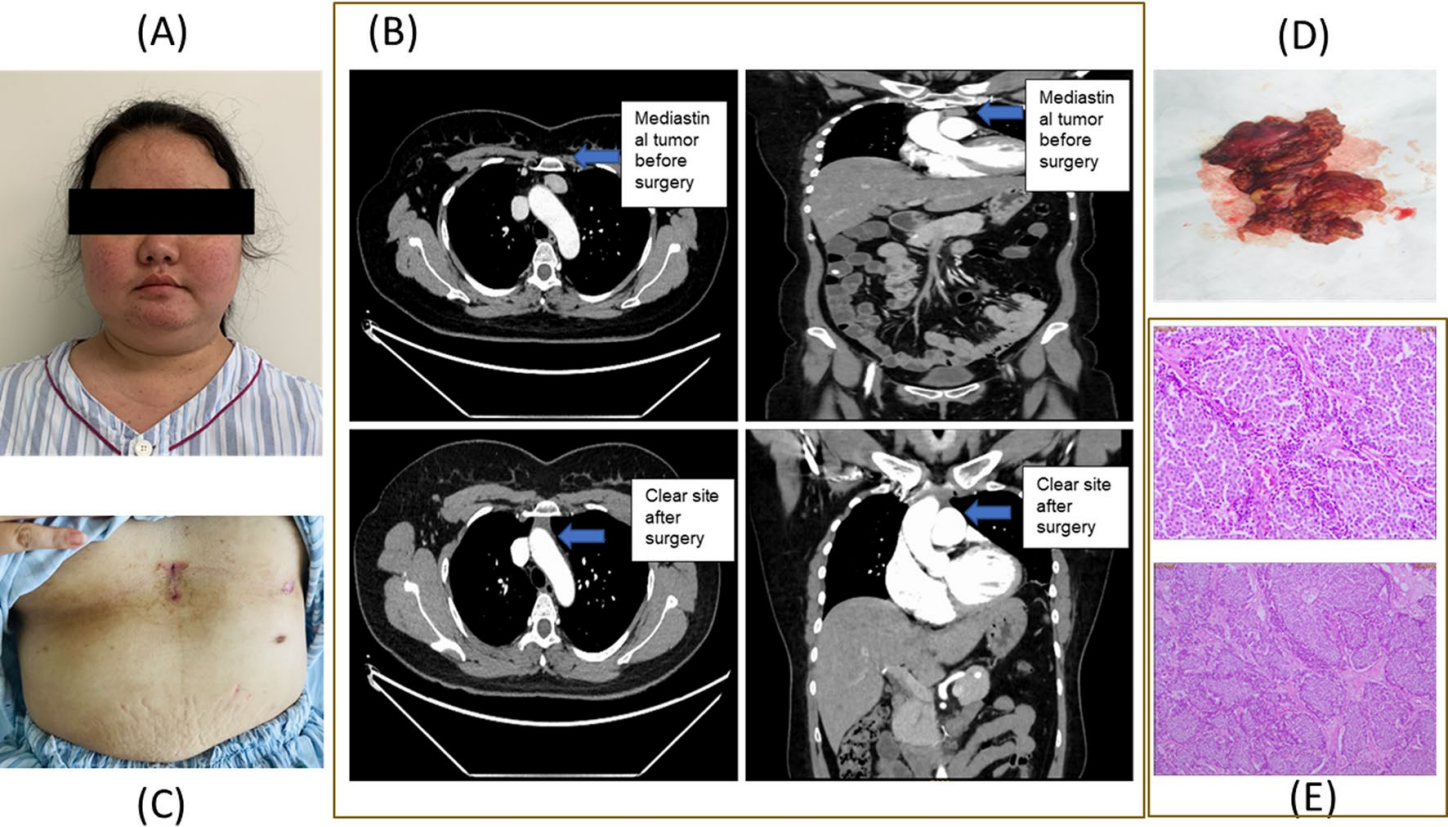
**A** Characteristic facial features of Cushing syndrome. **B** Preoperative (up) and postoperative CT scans (Down). **C** Scar via subxiphoid approach. **D** resected mass. **E** Histological morphology and immunohistochemical images confirmed neuroendocrine tumor (carcinoid)

Physical examination revealed hypertension (blood pressure: 141/100 mmHg) but no abnormalities of the lungs or heart. Initial vital indicators included 118 bpm heart rate, 20 bpm breathing rate, and 36.2 ^o^C body temperature. The BMI was 25.18 kg/m^2^, and the waist-hip ratio was 0.98. The abdomen has a little bloated appearance. She had the face of a full moon, the back of a buffalo, and central obesity (Fig. 1A). Except for the appearance of acne on both sides of the cheeks and jaws and facial hair, the patient did not have obvious hairiness or pigmentation. The rest of the physical examination was unremarkable.

### Laboratory examinations

On admission laboratory findings revealed K = 2.62 mmol/L (normal ranges: 3.50–5.10 mmol/L), HCO_3_ = 30.5 mmol/L (normal ranges: 22.0–27.0 mmol/L), and Na 147.1 mmol/L (normal ranges: 137.0-145.0 mmol/L). She had a WBC count of 8.56 × 10^9^/L (normal ranges: 3.50–9.50 × 10^9^/L). The seven items of regular stool testing + occult blood and tumor markers revealed no apparent abnormalities. Sinus tachycardia electrocardiogram (ECG) revealed ST-T alterations consistent with hypokalemia/alkalemia. The fasting glucose level was determined using an oral glucose tolerance test (OGTT); the glycated hemoglobin level was 6.3% (normal ranges: 4.0–6.0%), and all three diabetes autoantibodies were negative. Hormonal tests to determine the cause of hypertension are listed in Table 1. The presence of hypertension, hypokalemia, and alkalinity suggested hyperaldosteronism or CS [5].

**Table 1:** Hormonal tests to determine the cause of hypertension

Parameter	Lying down	Stand up
Angiotensin I (37 °C) ng/mL	2.6	2.61
Angiotensin I (4 °C) ng/mL	1.04	0.97
Renin activity (PRA) ng/mL/h	1.56	1.64
Angiotensin II pg/mL	132.59	185.53
Aldosterone (ALD) pg/mL	85.05	93.59
ARR (ALD/PRA)	5.45	5.71

CS detection was carried out by measuring 25-hydroxy vitamin D, which was found to be 21.3 ng/mL (20.1–30.0 ng/mL/vitamin D deficiency). Hormonal examinations showed that 17-hydroxyprogesterone was 22.54 ng/mL (normal ranges: 0.30–2.34 ng/mL), dehydroepiandrosterone sulfate was 799.50 µg/dL (normal ranges: 98.80–340.00 µg/dL), androstenedione was more than 10 ng/ml (normal ranges: 0.30–3.30 ng/ml), anti-Müllerian hormone was 11.64 ng/ml (normal ranges: 2.80–6.30 ng/mL), 17-ketocorticosteroid was 88.3 mg/24 h (normal ranges: 6.0–25.0 mg/24 h), and 17-hydroxycorticosteroid was 63.4 mg/24 h (normal ranges: 2.0–10.0 mg/24 h). Morning cortisol levels were increased to 1750 nmol/L (morning normal ranges : 133.0–537.0 nmol/L), ACTH levels were increased to 57.5 pmol/L (normal ranges: 1.60–13.90 pmol/L). Because cortisol levels increased as a result of disruption of circadian rhythm, a dexamethasone suppression test was performed. The morning cortisol level was not suppressed to < 50 nmol/l after the low dose (2 mg) dexamethasone suppression test, while ACTH remained substantially elevated, suggesting ACTH-dependent CS. There were no apparent abnormalities with respect to urine vanilla mandelic acid, urine catecholamines, thyroid function, parathyroid hormone, IGF-1, growth hormone, or gonadotropin. The ambulatory blood pressure was 196/117 mmHg, with a maximum systolic blood pressure of 227 mmHg, a maximum diastolic blood pressure of 127 mmHg, a minimum systolic blood pressure of 178 mmHg, and a minimum diastolic blood pressure of 101 mmHg. The mineral density of the bones was normal.

### Imaging examinations

Detailed imaging tests were performed to identify the underlying pathology. In the renal arteries, kidneys, and adrenal glands, color Doppler ultrasound did not reveal apparent abnormalities. The brain computed tomography (CT) scan and magnetic resonance imaging (MRI) revealed no pituitary adenoma. A chest CT scan revealed inflammation in the posterior basal region of the right lower lobe. The superior anterior mediastinum was characterized by round-shaped isodensity lesions with distinct borders (Fig. 1B). The maximum dimension was 16 mm × 24 mm. The CT value of the lesion on the plain scan was about 43 HU, whereas the CT value of the enhanced scan was around 88HU in the arterial phase and approximately 107 HU in the venous. The remainder of the mediastinum was clear. The images of the lungs, pancreas, gallbladder, and liver were normal. The CT scan of the adrenals revealed widespread adrenal hyperplasia on both sides, as well as left kidney stones. There were no apparent abnormalities in the form, size, or density of the uterus. There was no effusion in the pelvis and no enlargement of the pelvic lymph nodes. There was no pelvic effusion or swelling of the pelvic lymph nodes. Colon polyps pathology with serrated polyps was observed during gastrointestinal endoscopy.

The presence of masses in the anterior superior mediastinum soft group led us to suspect thymoma. Other noteworthy imaging findings were diffuse hyperplasia of both adrenal glands and multiple small stones in both kidneys. Based on biochemical and radiologic tests, the cause of her CS was suspected to be ectopic ACTH production [9, 10, 13].

## Final diagnosis

The final diagnosis of the presented case was CS due to ectopic ACTH production.

## Treatment

Minimally invasive thoracoscopic surgery via subxiphoid single-incision (Fig. 1C) was performed to remove the thymus tumor. The diagnosis of a neuroendocrine tumor has been confirmed by histopathological examination. The diagnosis of pheochromocytoma was ruled out, and thyroid color Doppler ultrasound and calcitonin tests ruled out medullary thyroid cancer. No obvious surgical contraindications were found, so under general anesthesia, tracheal intubation, thoracoscopic anterior mediastinal tumor resection, along with enlarged thymus resection were performed. A nodule-shaped structure with a size of 3.0 × 2.5 × 1.2 cm was incised and removed. The cut surface was solid, gray, and fine in texture, and there was no special adipose tissue (Fig. 1D). The tumor was diagnosed as a thymic carcinoid considering the symptoms, laboratory, imaging, and pathological results of the patient.

### Histology examination

Histological morphology and immunohistochemical results revealed that it was a carcinoid neuroendocrine tumor (G1) (Fig. 1E).

### Outcome and follow-up

**Fig. 2:**
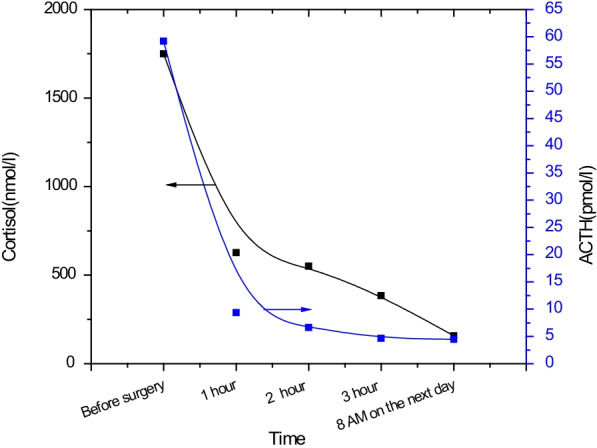
Changes in cortisol and ACTH levels after surgery

Compared to its preoperative value, cortisol level was reduced to half of the levels 1 h after surgery, and at 8 o’clock on the first day postoperatively, the levels reduced to 1/10 of preoperative value, and ACTH levels also showed a significant reduction, which tends to normalize after surgery (Fig. 2). A postoperative CT (7 days after the surgey) is shown in Fig. 1B, which indicated that anterior mediastinal space occupation changes, and bilateral adrenal glands showed no obvious lesions. No abnormal tissue density and space-occupying lesions were found. The trachea and bronchi were unobstructed and there was no sign of stenosis or obstruction. The mediastinum structure was clear, no space-occupying lesions were seen, and no enlarged lymph nodes were seen near the trachea, subcarinal anterior blood vessels, and posterior to the vena cava.

After treatment for anti-infection, phlegm, analgesic, hypoglycemic, antihypertensive and fluid supplementation, the patient’s blood sugar became normal and blood pressure was controlled. Surgical treatment also helps in changing from moon shape face to slim face and healing of wounds. The patient was recovered very well and did not have any other uncomfortable symptoms at the time of discharge (10 days after the surgery). The surgical incision below the xiphoid has healed absolutely then (Fig. 1C).

## Discussion

CS is a rare endocrine disorder, presenting a cluster of symptoms caused by an excessive level of glucocorticoids [14]. It is often clinically noticeable; however, in some instances, the spectrum is wide and nonspecific, coinciding with several medical disorders such as type 2 diabetes, obesity, and PCOS [15]. We present a unique case of CS due to a small tumor detected in the anterior mediastinum, which was successfully managed by a novel minimally invasive approach.

Clinical features of CS include violaceous striae, muscle weakness, bruisability, and bone loss [16]. However, in our case, osteoporosis and muscular weakness were not apparent. It should be emphasized that in CS, the severity of symptoms is determined by the degree of hypercortisolemia rather than the duration of exposure or tumor size [17]. Hypercortisolemia can be produced by a number of factors, the most common of which is ACTH secretion induced by pituitary or non-pituitary tumors. Glucocorticoids have been shown to activate aldosterone target cells’ mineralocorticoid receptors (MR) [18]. In episodes of ectopic ACTH production, MR activation can result in severe electrolyte imbalances such as hypernatremia and metabolic alkalosis. In our case, a small neuroendocrine tumor in the anterior superior mediastinum soft group was found to cause the severe hypercortisolism of the patient.

Neuroendocrine tumors can occur in multiple organs and tissues [19], while gastrointestinal or lung tumors are the most common; although abnormal mediastinal neuroendocrine tumors have been reported, their exact origin and classification remain uncertain [20]. These tumors exhibit an aggressive response in conjunction with common endocrinopathies. According to a recent study on ectopic CS caused by thymic neuroendocrine tumors, fewer than 20% of such patients just had a single tumor in mediastinum at the time of presentation [14]. In addition, 88% of the patients with mediastinal tumors had surgery, and histological subtypes of the tumors were unusual in almost half of the cases [14]. In our case, at the time of presentation, the differential diagnosis included CS and hyperaldosteronism.

ACTH may be secreted by thymic carcinoid and thyroid medullary cancer [21]. In our patient, thyroid abnormalities were also less probable because of adequate thyroid function tests and negative indications for hyper/hypothyroidism. The diagnosis of medullary thyroid cancer was also ruled out based on ultrasound data. These findings suggest that, in our case, ACTH secretion was independent of hypothalamic-pituitary axis regulation [13, 22].

Although the clinical signs and symptoms of CS are minor, metabolic abnormalities are more noticeable. Importantly, hypokalemia can be detected in up to 58% of people [23]. Our patient was hypertensive and exhibited significant electrolyte abnormalities, both of which were consistent with high ectopic ACTH secretion [5]. The patient’s morning cortisol levels, as well as ACTH levels, were determined to be significantly increased. In our case, high-dose dexamethasone did not diminish cortisol or ACTH levels, confirming ectopic production.

Despite extensive research, the pathogenesis of thymomas and the best treatment options are still open for discussion [24]. Removal of all the involved tissue is the main aim in the treatment of thymic tumors. With minimally invasive thoracic surgery under the subxiphoid approach, we have obtained an outstanding result. Previous studies have described the thoracoscopic treatment of apical bullous disease and excision of peripheral pulmonary nodules. Non-operatively, it is difficult to determine the clinical stage of the thymomas and the size of the resection required to manage the tumor.

In essence, our case indicates that if there are no metastases, thoracoscopic resection of the whole lesion can be performed. According to our previous surgical experience, minimally invasive thoracoscopic surgery via a single subxiphoid incision has less trauma and can shorten the operation time. It can also greatly reduce the postoperative pain of the patients and make the postoperative recovery faster compared with the surgical resection of the thymic tumor through traditional lateral thoracic wall incision. In addition, thoracoscopic surgery via a single subxiphoid incision can remove the thymic tumor in a larger area, sweep the adipose tissue around the thymus more thoroughly, and has no significant increase in surgical complications. However, if preoperative CT confirms a huge mediastinal tumor or local invasion, thoracoscopic excision of the tumor via the subxiphoid incision would not be a preferred treatment option. A more intensive surgical approach is desired via thoracotomy or sternotomy in the case of advanced disease.

## Conclusions

Thymic carcinoids are uncommon; nonetheless, if an ectopic Cushing syndrome diagnosis is suspected, these lesions should be investigated, and a chest radiograph followed by CT should be performed to screen for this critical source of ectopic ACTH production that leads to CS. Rapid resolution of hypercortisolemia in our case suggests that thoracoscopic resection is an effective surgical therapy for such patients. If the surgical guidelines are followed correctly, thoracoscopic surgical excision of the thymic tumor by a subxiphoid incision would be an advanced and safe surgical procedure.

## Data Availability

The datasets used and/or analyzed during the current study are available from the corresponding author on reasonable request.

## Abbreviations

CS: Cushing syndrome
ECS: Ectopic CS
ACTH: Adrenocorticotropic hormone
PCOS: Polycystic ovary syndrome
ECG: Electrocardiogram
OGTT: Oral glucose tolerance test
CT: Computed tomography
MRI: Magnetic resonance imaging
